# Nutrient and antinutrient composition of yellow yam (*Dioscorea cayenensis*) products

**DOI:** 10.1016/j.dib.2017.02.022

**Published:** 2017-02-16

**Authors:** Oladejo Thomas Adepoju, Oluwatosin Boyejo, Paulina Olufunke Adeniji

**Affiliations:** a Department of Human Nutrition, Faculty of Public Health, College of Medicine, University of Ibadan, Ibadan, Nigeria; b Department of Hospitality and Tourism Management, Redeemer׳s University, Ede, Osun State, Nigeria

**Keywords:** Yellow yam, Processing methods, Nutrient retention, Antinutrients

## Abstract

The data presented in this article are related to research article titled “Effects of processing methods on nutrient and antinutrient composition of yellow yam (*Dioscorea cayenensis*) products” (Adepoju et al., 2016) [1]. This article documented information on nutrient and antinutrient composition as well as nutrient retention of *Dioscorea cayenensis* products. Fresh *Dioscorea cayenensis* tubers obtained from Bodija market were prepared into raw sample and local delicacies and analysed for proximate, mineral, vitamin and antinutrient composition using AOAC methods [2]. Data obtained were analysed using ANOVA, and level of significance set at p<0.05. Processing significantly improved macronutrients and energy content of yam products, and led to significant reduction in values of all antinutrient content of the products (p<0.05).

**Specifications Table**

**Table:** 

Subject area	Food Analysis
More specific subject area	Nutrient composition
Type of data	Tables
How data was acquired	Chemical Analysis of yam sample using Air oven, micro-Kjeldahl apparatus, Soxlhet extractor, ballistic bomb calorimeter, flame photometer, Atomic absorption spectrophotometer, and spectrophotometric methods
Data format	Analysed
Experimental factors	Nutrient and antinutrient composition and nutrient retention of yellow yam products
Experimental features	AOAC methods were used for proximate composition, Flame emission and Atomic absorption spectrometric methods were used for minerals determination, while spectrophotometric methods were used for vitamin and antinutrient determinations.
Data source location	Ibadan, Oyo State, Nigeria
Data accessibility	The data is available in this article

**Value of the data**

• Important for estimating nutrient intakes and contribution from yellow yam products
• Can assist Dietitians in calculating portion sizes for their clients
• Can be included in the Food Composition Database as reference for food composition compilers and comparison of recipe formulation of other food products.
• Can popularize and improve consumption of yellow yam products as means of meeting nutrient requirements and improving dietary diversity of its consumers.

## 1 Data

The data provide useful information on nutrient and antinutrient content, and nutrient retention of yellow yam products to enable consumers, nutritionists and Dietitians estimate nutrient contribution from yellow yam products. Table 1, Table 2, Table 3, Table 4 provide information on proximate nutrient composition, minerals, vitamins and antinutrient components of yellow yam respectively [1].

## 2 Experimental design, materials and methods

Fresh yam purchased from Bodija market in Ibadan, Oyo State, Nigeria was peeled and part of it used as raw yam (Sample 1). The second portion was roasted unpeeled with coal fire, scraped with knife and labelled Sample 2. The rest of the fresh peeled yam was cut into small pieces, washed with distilled water, and then divided into five portions. One portion was sliced into smaller pieces, washed with distilled water and drained. One portion of drained peeled yam was fried with vegetable oil (Sample 3). A second portion was boiled to dryness with distilled water at 100 °C and divided into two smaller portions treated as follows: the first smaller portion served as boiled yam (Sample 4), while the remaining smaller portion was prepared to porridge (Sample 5). The third portion of the peeled, sliced raw yam was washed with distilled water and boiled at 100 °C for 30 min, and then divided into two sub-portions. The first sub-portion was pounded with mortar and pestle with ordinary water (Sample 6), while the second sub-portion was pounded with the water used in cooking the yam (Sample 7) [3].

The fourth portion of the peeled, washed and sliced raw yam was washed with distilled water, mashed with a warring blender with addition of distilled water and mixed with 1 g of salt and 5 g of powdered dry pepper. The slurry paste obtained was moulded into balls and fried with vegetable oil to produce fried yam cake (*Ojojo*), (Sample 8). The fifth portion of peeled washed and sliced raw yam was parboiled at 60 °C for 10 min, left overnight in the warm water (18 h), drained and sun dried for 4 days. The dried parboiled yam was then grinded to powder, prepared with boiling water to form a thick paste called *Amala* (Sample 9) [4]. Chemical analyses were carried out using AOAC (2005) methods [2].

## Funding sources

This work was an M.Sc. project of Boyejo Oluwatosin carried out without any funding source.

## Figures and Tables

**Table 1:** Proximate nutrient composition of fresh and processed (As consumed) yellow yam Products (g/100g).⁎

Sample	MC	CP	CL	CF	Ash	CHO	G E
1	66.79±0.44	2.62±0.05	0.27±0.01	0.17±0.01	0.63±0.02	29.52±0.82	108.26±0.03
2	57.85±2.31	3.02±0.15	0.46±0.02	0.20±0.02	1.43±0.06	37.04±1.66	144.81±0.15
3	46.88±1.82	6.25±0.12	7.12±0.18	0.42±0.01	2.07±0.07	37.26±1.79	208.51±0.05
4	69.50±0.20	2.18±0.04	0.27±0.01	0.13±0.01	1.39±0.03	26.53±0.20	100.48±0.07
5	69.20±0.20	6.37±0.31	4.76±0.18	0.30±0.02	2.29±0.09	17.08±0.09	100.32±0.07
6	75.37±0.47	1.62±0.01	0.19±0.01	0.08±0.01	1.07±0.01	21.67±0.20	80.77±0.35
7	75.39±0.47	1.73±0.03	0.19±0.01	0.19±0.53	1.11±0.01	21.19±0.20	81.89±0.30
8	54.09±2.09	6.83±0.58	8.59±0.65	0.53±0.04	3.03±0.22	26.93±0.25	196.49±0.20
9	68.63±0.29	1.21±0.05	0.04±0.01	0.07±0.01	0.41±0.03	29.64±0.22	106.30±0.49

MC=Moisture Content, CP=Crude Protein, CL=Crude Lipid, CF=Crude Fibre, CHO=Carbohydrates, GE=Gross Energy.

Sample 1: Raw yam, Sample 2: Roasted yam, Sample 3: Fried yam, Sample 4: Boiled yam, Sample 5: Porridge, Sample 6: Pounded yam with ordinary water, Sample 7: Pounded yam with cooking water, Sample 8: *Ojojo*, Sample 9: *Amala.*

⁎ Values are means±SD of triplicate determinations.

**Table 2:** Mineral composition of fresh and processed (As consumed) yellow yam products (mg/100g).⁎

Sample	Na	K	Ca	Mg	P	Fe	Zn
1	8.53±0.05	262.30±0.25	22.53±0.13	61.53±0.25	19.50±0.10	0.79±0.02	0.39±0.01
2	6.36±0.09	294.59±0.12	17.79±0.15	10.92±0.14	26.99±0.16	0.63±0.01	0.24±0.02
3	6.55±0.11	326.99±0.14	12.74±0.14	9.29±0.11	22.74±0.14	0.59±0.02	0.18±0.02
4	4.86±0.07	217.81±0.08	13.68±0.05	8.48±0.05	19.95±0.10	0.47±0.01	0.22±0.06
5	12.36±0.08	293.15±0.05	22.05±0.10	18.95±0.09	23.10±0.13	1.04±0.01	0.74±0.01
6	4.03±0.52	173.01±0.06	9.98±0.05	6.58±0.07	15.75±0.07	0.35±0.01	0.16±0.01
7	3.78±0.07	176.09±0.07	10.92±0.07	6.81±0.09	6.81±0.09	0.37±0.03	0.17±0.01
8	17.19±0.09	428.03±0.31	32.24±0.25	27.51±0.12	33.40±0.22	1.52±0.02	1.07±0.02
9	9.53±0.31	206.53±0.35	23.43±0.25	24.80±0.30	28.83±0.30	1.02±0.03	0.19±0.02

⁎ Values are means±SD of triplicate determinations.

**Table 3:** Selected vitamin composition of fresh and processed (As consumed) yellow yam products (mg/100g).⁎

Sample	β-carotene (µg/)	Thiamine	Riboflavin	Ascorbic acid
1	7.65±0.03	0.14±0.01	0.06±0.01	14.38±0.03
2	11.32±0.02	0.00±0.00	0.00±0.00	2.13±0.01
3	31.20±0.02	0.00±0.00	0.00±0.00	2.07±0.02
4	7.53±0.01	0.01±0.00	0.00±0.00	1.44±0.01
5	40.67±0.01	0.07±0.01	0.05±0.01	3.76±0.01
6	5.13±0.01	0.00±0.00	0.00±0.00	1.09±0.01
**7**	5.61±0.01	0.00±0.00	0.00±0.00	1.15±0.01
8	95.21±0.02	0.12±0.01	0.09±0.01	3.81±0.02
9	6.18±0.03	0.05±0.01	0.00±0.00	3.27±0.02

⁎ Values are means±SD of triplicate determinations.

**Table 4:** Antinutrient composition of fresh and processed (As consumed) yellow yam Products (mg/100g).⁎

Sample	Phytate	Oxalate	Tannin	Saponin
1	0.05±0.00	0.48±0.00	0.01±0.00	0.06±0.00
2	0.05±0.00	0.39±0.01	0.01±0.00	0.05±0.00
3	0.03±0.00	0.34±0.00	0.00±0.00	0.03±0.00
4	0.04±0.00	0.38±0.00	0.01±0.00	0.04±0.00
5	0.02±0.00	0.13±0.00	0.00±0.00	0.02±0.00
6	0.04±0.00	0.36±0.00	0.01±0.00	0.03±0.00
7	0.04±0.00	0.37±0.00	0.01±0.00	0.04±0.00
8	0.03±0.00	0.12±0.00	0.00±0.00	0.03±0.00
9	0.02±0.00	0.11±0.00	0.00±0.00	0.01±0.00

⁎ Values are means±SD of triplicate determinations.
